# USP22 Induces Cisplatin Resistance in Lung Adenocarcinoma by Regulating γH2AX-Mediated DNA Damage Repair and Ku70/Bax-Mediated Apoptosis

**DOI:** 10.3389/fphar.2017.00274

**Published:** 2017-05-17

**Authors:** Aman Wang, Zhen Ning, Chang Lu, Wei Gao, Jinxiao Liang, Qiu Yan, Guang Tan, Jiwei Liu

**Affiliations:** ^1^Department of Oncology, The First Affiliated Hospital, Dalian Medical UniversityDalian, China; ^2^Department of Hepatobiliary Surgery, The First Affiliated Hospital, Dalian Medical UniversityDalian, China; ^3^City College, Zhejiang UniversityHangzhou, China; ^4^Department of Thoracic Surgery, Zhejiang Cancer HospitalHangzhou, China; ^5^Department of Biochemistry and Molecular Biology, Dalian Medical UniversityDalian, China

**Keywords:** ubiquitin-specific peptidase 22, DNA damage repair, apoptosis, cisplatin resistance, lung adenocarcinoma

## Abstract

Resistance to platinum-based chemotherapy is one of the most important reasons for treatment failure in advanced non-small cell lung cancer, but the underlying mechanism is extremely complex and unclear. The present study aimed to investigate the correlation of ubiquitin-specific peptidase 22 (USP22) with acquired resistance to cisplatin in lung adenocarcinoma. In this study, we found that overexpression of USP22 could lead to cisplatin resistance in A549 cells. USP22 and its downstream proteins γH2AX and Sirt1 levels are upregulated in the cisplatin- resistant A549/CDDP cell line. USP22 enhances DNA damage repair and induce cisplatin resistance by promoting the phosphorylation of histone H2AX via deubiquitinating histone H2A. In addition, USP22 decreases the acetylation of Ku70 by stabilizing Sirt1, thus inhibiting Bax-mediated apoptosis and inducing cisplatin resistance. The cisplatin sensitivity in cisplatin-resistant A549/CDDP cells was restored by USP22 inhibition *in vivo* and *vitro*. In summary, our findings reveal the dual mechanism of USP22 involvement in cisplatin resistance that USP22 can regulate γH2AX-mediated DNA damage repair and Ku70/Bax-mediated apoptosis. USP22 is a potential target in cisplatin-resistant lung adenocarcinoma and should be considered in future therapeutic practice.

## Introduction

Non-small cell lung cancer (NSCLC), which comprises 80 to 85% of all lung cancers, is the most fatal cancer worldwide, with a 5-year survival as low as 13% (Molina et al., 2008; Siegel et al., 2015). Currently, platinum-based doublet chemotherapy regimens are the most standard first-line treatment strategy for advanced NSCLC, especially in patients without driver gene mutations (Zappa and Mousa, 2016). However, the efficacy of standard first-line chemotherapy is only approximately 30%, and resistance to platinum-based chemotherapy including cisplatin is one of the most important reasons for treatment failure and poor prognosis in NSCLC (Fennell et al., 2016). It is well known that cisplatin acts on multiple cellular targets representing diverse signal transduction pathways (Rose et al., 2014). The mechanism underlying cisplatin resistance is extremely complex and multi-factorial. Therefore, further elucidation of molecular mechanisms underlying cisplatin resistance would improve the efficacy and prognosis of NSCLC.

Cisplatin is a cell-cycle non-specific cytotoxic drug containing a bifunctional group similar to those of alkylating agents. It binds to nucleophilic groups in the cell and is distributed non-selectively in tumor tissues. The molecular mechanisms of cisplatin include binding of the drug to DNA and non-DNA targets to form a variety of cross links and monoadducts, which contribute to the cytotoxicity by blocking DNA replication and inducing apoptosis (Galluzzi et al., 2012). Previous results have demonstrated that the reduced cellular accumulation of cisplatin, inactivation through covalent reaction with intracellular thiol-containing molecules, enhanced DNA repair processes and failure of the apoptotic pathway maybe the major factors of cisplatin resistance (Galluzzi et al., 2014).

Ubiquitin-specific peptidase 22 (USP22) is a newly discovered member of the deubiquitinase (DUB) family, and it is involved in many biological processes, including tumor development, cell growth and differentiation, cell cycle regulation, transcriptional activation and signal transduction (Melo-Cardenas et al., 2016). USP22 is moderately expressed in a variety of normal human tissues, such as heart and skeletal muscle, but weakly expressed in lung and liver (Lee et al., 2006). Recently, overexpression of USP22 has been reported in several solid tumors including NSCLC and demonstrated to be associated with poor prognosis (Liu et al., 2010, 2011; Yang et al., 2011; Zhang et al., 2011). As a subunit of hSAGA, USP22 participates in the deubiquitination of histones H2A and H2B and the acetylation of histone H4 to regulate gene transcription and expression (Zhang et al., 2008). H2AX is a subtype of histone H2A. In the process of DNA double-strand breaks (DSBs) repair, the H2AX 139^Ser^site is phosphorylated. Histone H2AX phosphorylation is a sensitive marker for DNA DSBs and associated with resistance to multiple chemotherapy drugs including cisplatin. In addition, certain non-histone proteins such as sirtuin 1 (Sirt1) and fructose-bisphosphatase 1 (FBP1) could be deubiquitinated by USP22 (Atanassov and Dent, 2011; Lin et al., 2012; Ao et al., 2014). Sirt1 deacetylates a variety of proteins including P53 and Ku70, and participates in the processes of proliferation, apoptosis, DNA damage repair and drug resistance in tumor cells (Jeong et al., 2007; Shin et al., 2014). USP22 can deubiquitinate and stabilize the expression of Sirt1. Thus, USP22 may be ascribed a major role in chemotherapy resistance in tumor cells. The relationship between USP22 and cisplatin resistance has not been reported.

This study focused on the relationship between USP22 and cisplatin resistance in lung adenocarcinoma and explored the potential mechanisms to provide a new therapeutic target for reversing cisplatin resistance.

## Materials and Methods

### Cell Lines and Cell Culture

The human lung adenocarcinoma cell line A549, HEK293T (obtained from cell bank of the Committee on Type Culture Collection of the Chinese Academy of Sciences, Shanghai, China) and cisplatin-resistant variant cell line A549/CDDP (obtained from Cancer Hospital Chinese Academy of Medical Sciences, Beijing, China) were cultured in DMEM medium (Life Technologies). Cells were cultured in a 1:1 mixture of Dulbecco’s modified Eagle’s medium (DMEM) supplemented with 10% fetal bovine serum (Gibco, Gran Island, NY, United States). Moreover, 0.066 μM cisplatin was contained in the A549/CDDP cell medium to keep its drug-resistant phenotype. The subsequent experiments will use cells in the logarithmic phase of growth.

### Plasmid Preparation and Cell Transfection

The USP22 overexpression lentiviral plasmids and USP22-shRNA were designed and synthesized by Shanghai GenePharma Co., Ltd (Shanghai, China). Lentiviral infection was performed as described. HEK293T cells were transfected with either PLOC-vector, PLOC-USP22, Sh-control (5′-TTCTCCGAACGTGTCACGT-3′) or USP22-shRNA. After 48 h of transfection, the medium containing retroviruses was collected, filtered, treated by polybrene (1 μg/ml) and transferred to A549 target cells. Infected cells were selected with puromycin (2.5 μg/ml) for 14 days and two individual clones were isolated. Three pairs of shRNA named USP22-shRNA-1, USP22-shRNA-2 and USP22-shRNA-3. The target sequence of USP22-shRNA-3 was listed as follows: 5′-GCAAGGCCAAGTCCTGTATCT-3′.

### Transfection of siRNA

To assess the Sirt1 inhibition, 50 nM of Sirt1 siRNA (GenePharma, Shanghai, China) were transfected into A549-USP22, A549/CDDP-sh-control, or A549/CDDP-sh-USP22 cells by Lipofectamine 2000 based on manufacturer’s instructions. Cells transfected with the scrambled siRNA have been adopted as the negative control. The cells were collected 48 h after the transfection. “Three pairs of siRNA named Sirt1-siRNA-1, Sirt1-siRNA-2, and Sirt1-siRNA-3 were used. The sequences of Sirt1-siRNA-1, Sirt1-siRNA-2 and Sirt1-siRNA-3 were as follows: 5′-GUACAAACUUCUAGGAAUG-3′, 5′-GATGAAGTTGACCTCCTCA-3′, 5′-GCGAUUGGGUACCGAGAUA-3′.

### *In Vitro* Chemosensitivity Assay

The *in vitro* chemosensitivity of cisplatin-resistant and parental A549 cells to cisplatin was determined by CCK-8 assay. Briefly, cells have been seeded into 96-well plates (5 × 10^3^ cells/well) and hence to make it possible for overnight adherence. Subsequently, the cells were treated with various concentrations of cisplatin. Ten microliter of CCK-8 (Cell Counting Kit-8, C04-13; Dojindo Laboratories, Kumamoto, Japan) was put into each well after 24 or 48 h and was incubated for 4 h under 37°C. A microplate reader at 450 nM has been used to analyze the plates. Every experiment was done more than three times.

### Western Blot Assay

Proteins were separated by SDS-PAGE and transferred to nitrocellulose membrane (Bio-Rad, Hercules, CA, United States). Membranes were blocked in a buffer (TBS: 50 mM Tris-HCl, 150 mM NaCl, pH 7.4) containing 5% bovine serum albumin and 0.1% Tween-20, followed by incubation with the primary antibodys USP22 (ab4812, 1:2000, Abcam, Cambridge, UK), Sirt1 (2496, 1:2000, CST, United States), H2AX (2595, 1:1000, CST, United States), γ-H2AX (Ser 139)(9718, 1:1000, CST, United States), Ubi-H2A (Lys119) (8240, 1:1000, CST, United States), Ku70 (10723-1-AP, 1:1000, Proteintech, United States), Bax (50599-2-lg, 1:1000, Proteintech, United States), cytochrome C (10993-1-AP, 1:1000, Proteintech, United States) or GAPDH (10494-1-AP, 1:3000, Proteintech, United States). The immunoreactive proteins were visualized using the ECL western blotting system (Bio-Rad, Hercules, CA, United States), and densitometric analysis was performed using BioImaging systems (UVP, labworksTM, ver 4.6). Mean values of the data obtained from three separate chambers were presented.

### Immunohistochemistry

Tumor tissue specimens were fixed with 10% neutral formalin for 24–48 h and routinely processed for paraffin embedding. IHC staining was performed as reported previously (Ning et al., 2014). Sirt1, Ki-67, and γ-H2AX antibodies (1:200) were detected using the streptavidin–peroxidase conjugate method.

### Flow Cytometric Analysis of Cell Cycle and Apoptosis

Cells were plated in 6-well plates (2 × 10^5^ cells/well). Cells were treated by cisplatin with 0 or 0.33 μM. The propidium iodide stained the cells after 24 h. The BD Cycle Test Plus DNA Reagent Kit (BD Biosciences, Shanghai, China) has been used in the cell-cycle analysis, following the protocol offered by the manufacturer. The cells were analyzed by FAC scan (BD Biosciences, Shanghai, China), and the percentage of cells in G0/G1, S, or G2/M phase was estimated. Every experiment was conducted at least three times.

Cells were seeded in 6-well plates and resuspended in binding buffer, washed with PBS twice and trypsinized after 48 h. Then, Annexin V/PI (Invitrogen, United States) was used to stain the cells for 15 min in the dark at the room temperature. Then, cell population analysis was conducted by flow cytometry.

### Colony-Forming Assay

3 to 5 × 10^3^ cells were plated in triplicate in a 24-well plate. Twenty-four hours later, treatment was initiated. After 14 days, cells were fixed and stained with crystal violet. Quantification was done using Adobe Photoshop, a method described elsewhere. All P values were calculated using the Student’s *t*-test.

### Comet Assay

Slides were pre-coated with 1% normal melting point agarose. About 3 × 10^4^ cells were mixed with 70 μL of 1% low melting point agarose in phosphate-buffered saline (PBS), and rapidly spread onto the pre-coated slides. The slides were immediately placed in cold lysis buffer containing 2.5 M NaCl, 100 mM EDTA, 10 mM Tris (pH 7.5), 1% Triton X-100, 1% INCI, and 10% DMSO at 4°C for 1–3 h. The slides were then placed in the electrophoresis solution for 20 min to facilitate DNA unwinding before electrophoresis was conducted for 20 min at 25 V and 300 mA. After electrophoresis, the slides were washed with PBS and then stained with DAPI. The individual cells were viewed using an Olympus BX51 UV fluorescence microscope (Olympus, Japan).

### *In Vivo* Assays for Tumor Growth

The *in vivo* tumorigenesis assays were performed, as followed. A549/CDDP or A549/CDDP-sh-USP22 (2 × 10^6^) were injected into the left and right dorsal flank of 5-week-old female nude nice, respectively. At 1 week post-transplantation, cisplatin (3.5 mg/Kg) were given every three days through intra-peritoneal injection. Growth curves were plotted, based on mean tumor volume at each time point, for each experimental group. The tumor dimensions were measured every 3 days using a digital caliper. The tumor volume (mm^3^) was calculated as follows: *V* = ab^2^/2, where a and b are the largest and smallest tumor diameters measured at necropsy, respectively. After 35 days, the mice were killed and the tumor tissues were harvested for use in further experiments. The animal research protocols used in the present study were approved by the Animal Studies Committee of the Dalian Medical University.

### Statistical Analysis

All statistical analyses were done using the SPSS software package (version 13.0, SPSS Inc., Chicago, IL, United States). Data were shown as the mean ± SD of no less than three separate experiments. Bars in the graph represent standard deviation. Student’s *t*-test was used for comparing the difference between two groups and *p* < 0.05 was considered as statistically significant.

## Results

### Overexpression of USP22 Was Correlated with Acquired Resistance to Cisplatin in A549 Cells

**FIGURE 1:**
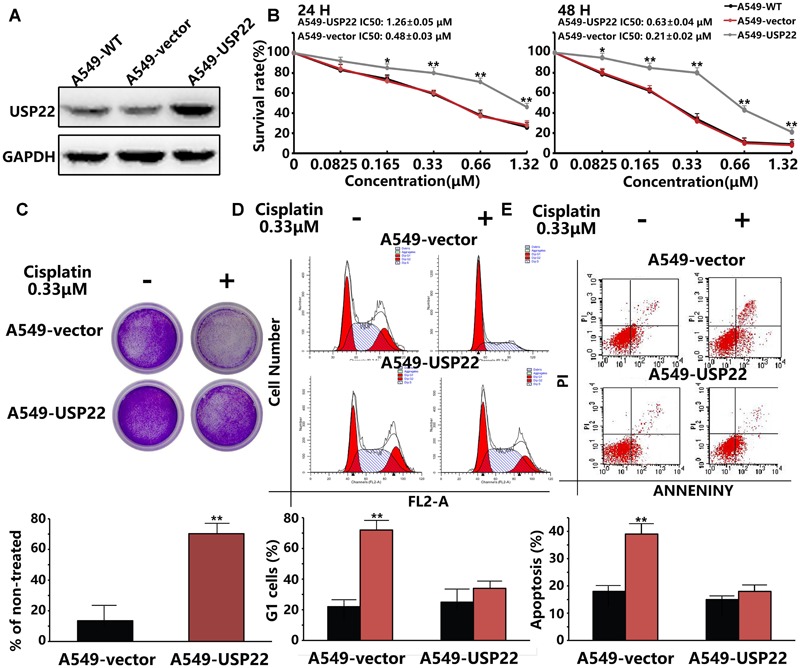
**Overexpression of USP22 was correlated with acquired resistance to cisplatin in A549 cells. (A)** Western blot analysis of USP22 expression in A549-WT, A549-vector, and A549-USP22 cells (the cell clones which were stably transfected with vector or USP22 overexpression plasmids). **(B)** The sensitivity to cisplatin of A549-WT, A549-vector, and A549-USP22 cells was detected by CCK-8. Cells were exposed to various concentrations of cisplatin for 24 and 48 h. **(C)** The response of A549-vector and A549-USP22 cells to cisplatin was tested by colony-forming assay. Cell lines were treated continuously with either 0 or 0.33 μM of cisplatin for 14 days. **(D)** A549-vector and A549-USP22 cells were treated with 0 or 0.33 μM of cisplatin for 48 h and subjected to flow cytometry analysis of cell cycle distribution. Percentage change in G1 phase is shown. **(E)** Cisplatin-induced apoptosis in A549-vector and A549-USP22 cells was demonstrated by flow cytometric analysis. Cell lines were treated continuously with either 0 or 0.33 μM cisplatin for 48 h. Every experiment was conducted at least three times, and the average is shown (mean ± SD). ^∗^*P* < 0.05, ^∗∗^*P* < 0.01, significant.

To validate the hypothesis that USP22 can promote cisplatin resistance in A549 cells, we stably overexpressed the USP22 cDNA in A549 cells (**Figure 1A**). Drug susceptibility was detected by cholecystokinin 8 (CCK8) and colony formation assays. The CCK8 results showed that the IC50 of 24h and 48h cisplatin treatment was 1.26 ± 0.05 μM and 0.63 ± 0.04 μM for A549-USP22 (A549 cells overexpressing USP22), which was 2.6 and 3.0-fold higher compared with the A549 cell line (**Figure 1B**), respectively. In the colony formation experiment, cells were plated at a low density and treated continuously with 0.33 μM of cisplatin for 14 days. The results showed that the number of colonies formed by A549-USP22 was 5.2-fold higher compared with the control cells (**Figure 1C**). These results provide evidence that overexpression of USP22 was correlated with acquired resistance to cisplatin in A549 cells.

We examined the G1-S arrest induced by cisplatin by flow cytometry. Cells were treated with 0.33 μM of cisplatin for 48 hours, and cell cycle profiles were examined. The control cells showed a significant increase in G1 phase cells, whereas the A549-USP22 cells did not show a significant G1 phase enrichment (**Figure 1D**). We also detected the apoptosis of treated cells by flow cytometry. The results showed that the apoptotic rate of A549-USP22 cells was significantly less than that of the control cells (**Figure 1E**). In summary, these results suggest that USP22 can overcome the cisplatin-induced G1-S arrest, the proliferation defect and apoptosis and allow cells to continue to grow in the presence of the drug.

### USP22 and Its Downstream Proteins γH2AX and Sirt1 Levels Are Upregulated in the Cisplatin-Resistant A549/CDDP Cell Line

**FIGURE 2:**
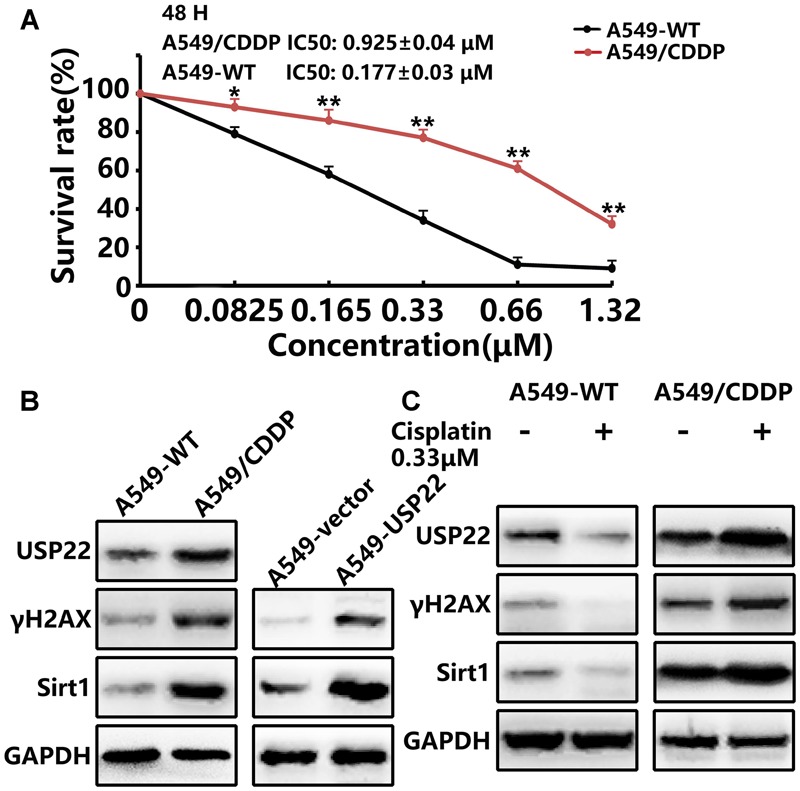
**USP22 and its downstream proteins γH2AX and Sirt1 levels are upregulated in the cisplatin-resistant A549/CDDP cell line. (A)** The sensitivity to cisplatin of A549-WT and A549/CDDP cells was detected by CCK-8. Cells were exposed to various concentrations of cisplatin for 24 and 48 h. **(B)** Western blot analysis of USP22, γH2AX and Sirt1 in A549-WT, A549/CDDP, A549-vector, and A549-USP22 cells. **(C)** Western blot analysis of the proteins of USP22, γH2AX and Sirt1 in A549-WT and A549/CDDP cells. Cell lines were treated continuously with either 0 or 0.33 μM cisplatin for 48 h. Every experiment was conducted at least three times, and the average is shown (mean ± SD). ^∗^*P* < 0.05, ^∗∗^*P* < 0.01, significant.

First, we utilized the CCK8 to detect the drug resistance of A549/CDDP cells. As shown in **Figure 2A**, the IC50 of 48 h cisplatin treatment for the drug-resistant A549/CDDP cell line was 0.925 ± 0.04 μM, which was 5.3-fold higher compared with the A549 cell line (0.177 ± 0.03 μM). Our results confirm that A549/CDDP cells showed increased resistance against cisplatin compared with parental cells.

Previous studies have confirmed the role of USP22 in regulating various processes in tumors, including DNA damage repair, proliferation and apoptosis. In this study, western blots indicated that the expression levels of USP22, γH2AX and Sirt1 in A549/CDDP were significantly higher than those in A549 cells. Meanwhile, the upregulation of γH2AX and Sirt1 was also found in the A549-USP22 cells (**Figure 2B**). To further clarify the roles of these proteins in cisplatin resistance, A549 and A549/CDDP cells were treated with 0.33 μM cisplatin. The results showed that, after cisplatin treatment of A549 cells, the USP22, γH2AX, and Sirt1 expression levels decreased significantly. Conversely, these proteins increased significantly after cisplatin treatment of A549/CDDP cells, compared with the untreated A549/CDDP cells (**Figure 2C**). These results confirm that USP22 is involved in the cisplatin resistance of A549/CDDP cells and H2AX, γH2AX, and Sirt1 may be responsible for USP22-mediated cisplatin resistance.

### USP22 Enhances DNA Damage Repair and Induces Cisplatin Resistance by Promoting the Phosphorylation of Histone H2AX via Deubiquitinating Histone H2A

**FIGURE 3:**
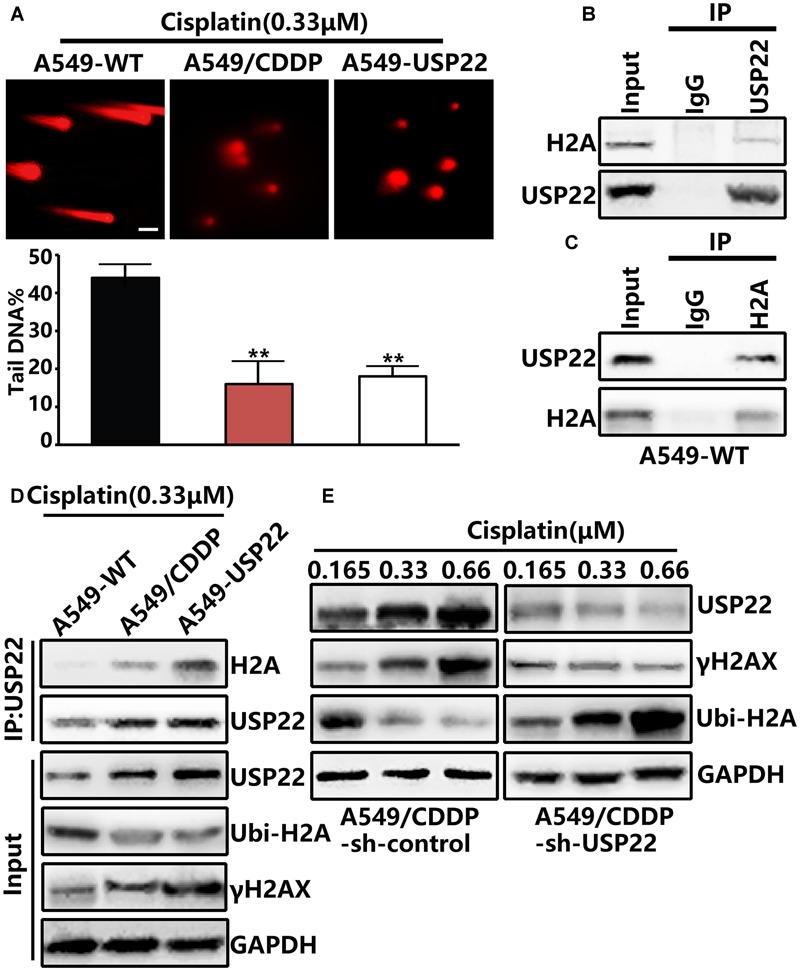
**USP22 enhances DNA damage repair and induce cisplatin resistance by promoting the phosphorylation of histone H2AX via deubiquitinating histone H2A. (A)** Comet assay was performed to assess DNA damage after 0.33 μM cisplatin treatment for 6 h in A549-WT, A549-USP22, and A549/CDDP cells (scale bar 20 μM). **(B,C)** H2A interacts with USP22 in A549-WT cells. A549-WT cells lysates were subjected to immunoprecipitation (IP) with control IgG, anti-USP22 **(B)**, and anti-H2A **(C)** antibodies. The immunoprecipitates were then blotted with the indicated antibodies. **(D)** H2A binding to USP22, γH2AX, and the ubiquitination of H2A (Lys119) was detected by IP and western blot after 0.33 μM cisplatin treatment for 48 h in A549-WT, A549/CDDP and A549-USP22 cells. **(E)** Western blot analysis of the proteins of USP22, γH2AX and Ubi-H2A after various concentrations of cisplatin treatment for 48 h. Every experiment was conducted at least three times, and the average is shown (mean ± SD). ^∗^*P* < 0.05, ^∗∗^*P* < 0.01, significant.

When DNA breaks occur in normal cells, the ataxia telangiectasia mutated (ATM) protein can activate DNA repair and direct histone H2AX to DSBs, leading to rapid histone H2AX phosphorylation to form γH2AX. To further clarify the mechanism, comet assays confirmed that after cisplatin treatment, the DNA migration distance of A549/CDDP and A549-USP22 cells decreased significantly compared with the control cells (**Figure 3A**), indicating that USP22 could significantly reduce the frequency of DNA DSBs induced by cisplatin. Subsequent immunoprecipitation (IP) experiments confirmed that USP22 bind to endogenous H2A in A549 cell line (**Figures 3B,C**). After 0.33 μM cisplatin treatment, the level of H2A binding to USP22 and γH2AX in A549/CDDP and A549-USP22 cells was significantly higher than that in the control cells, and the ubiquitination of H2A (Lys119) was lower than that in the control cells (**Figure 3D**). Three pairs of shRNA named USP22-shRNA-1, USP22-shRNA-2, and USP22-shRNA-3. Compared with control, the expression level of USP22 was only decreased successfully by USP22-shRNA-3. In addition, we stably inhibited the expression of USP22 in the A549/CDDP cell line (A549/CDDP-sh-USP22 cell line). In the case of A549/CDDP-sh-control cells being treated with different concentration of cisplatin, along with the concentration of cisplatin increased, USP22 and γH2AX level increased significantly and the ubiquitination of H2A (Lys119) downregulated simultaneously. Under the same treatment conditions, the results in A549/CDDP-sh-USP22 cells were contrary to A549/CDDP-sh-control cells (**Figure 3E**). The results confirm that USP22 could deubiquitinate H2A and promote the phosphorylation of histone H2AX, thus contributing to DNA damage repair and inducing cisplatin resistance in A549 cells.

### USP22 Decreases the Acetylation of Ku70 by Stabilizing Sirt1, thus Inhibiting Bax-Mediated Apoptosis and Inducing Cisplatin Resistance

**FIGURE 4:**
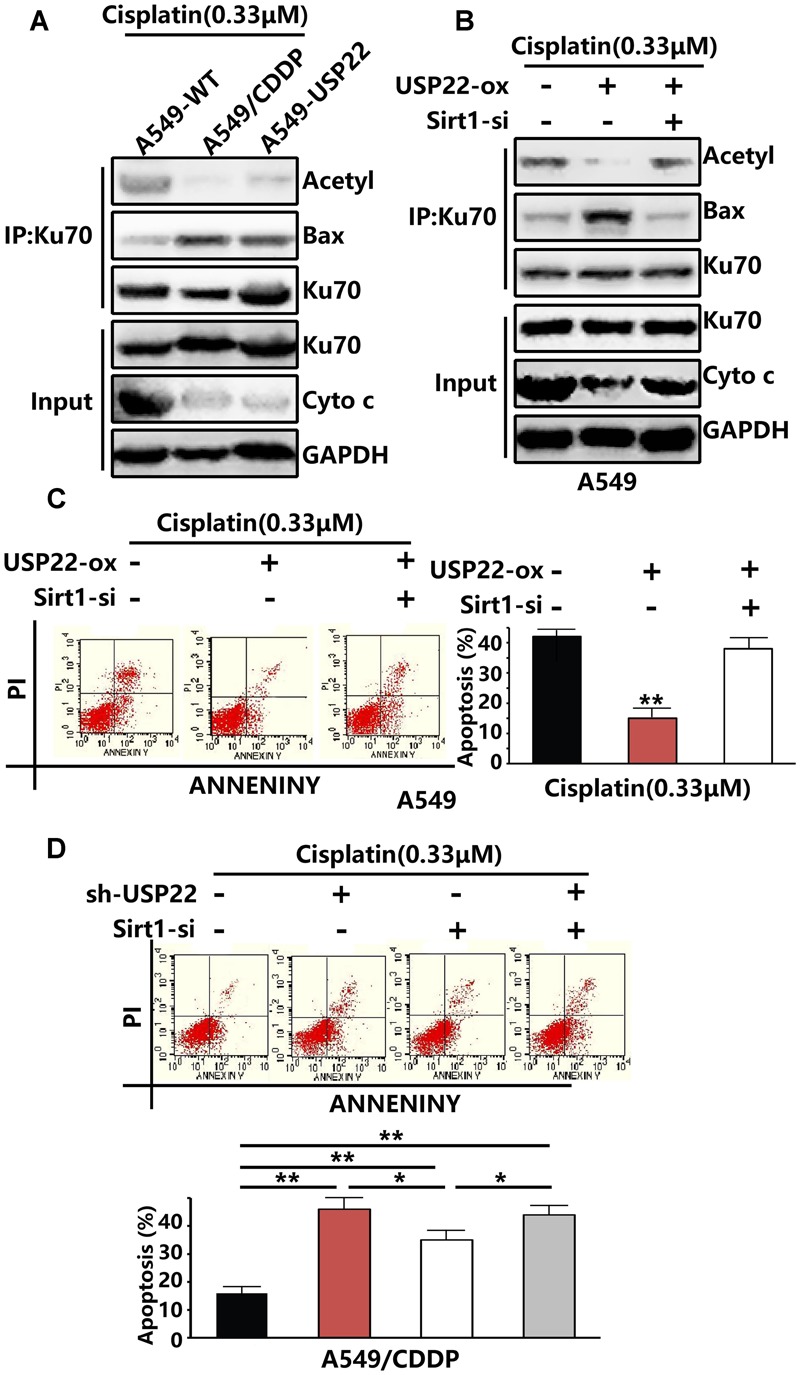
**USP22 decreases the acetylation of Ku70 by stabilizing Sirt1, thus inhibiting Bax-mediated apoptosis and inducing cisplatin resistance. (A)** Acetylated Ku70 and the Bax binding to Ku70 were detected by IP and cytochrome C was detected by western blot analysis in A549-WT, A549/CDDP and A549-USP22 cells, after 0.33 μM cisplatin treatment for 48 h. **(B)** Acetylated Ku70 and the Bax binding to Ku70 were detected by IP and cytochrome C was detected by western blot analysis in A549 cells which were transfected by vector, USP22, USP22+Sirt1-siRNA, after 0.33 μM cisplatin treatment for 48 h. **(C)** Apoptosis in A549 cells which were transfected by vector, USP22, USP22+Sirt1-siRNA was demonstrated by flow cytometric analysis, after 0.33 μM cisplatin treatment for 48 h. **(D)** Apoptosis in A549 cells which were transfected by si-control, sh-USP22, Sirt1-si and sh-USP22+Sirt1-si, after 0.33 μM cisplatin treatment for 48 h. Every experiment was conducted at least three times, and the average is shown (mean ± SD). ^∗^*P* < 0.05, ^∗∗^*P* < 0.01, significant.

Previous results showed that the expression levels of USP22 and Sirt1 in A549/CDDP were upregulated after cisplatin treatment (**Figure 2B**). Ku70 in the cytoplasm can bind to the Bax protein, thus preventing Bax from entering the mitochondria, inhibiting the release of cytochrome C and playing an endogenous anti-apoptotic role. Ku70 acetylation leads to the dissociation of Bax protein and Ku70, and Bax protein translocates to the mitochondria, leading to the initiation of apoptosis. Sirt1 has been shown to be able to downregulate Ku70 acetylation. IP experiments showed that, after treatment with 0.33μM of cisplatin, the level of Ku70 acetylation in A549/CDDP and A549-USP22 cells decreaed significantly compared with the control cells, whereas the level of Bax binding to Ku70 increased significantly compared with the control cells. Under the same treatment conditions, the cytochrome C expression level in A549/CDDP and A549-USP22 cells was significantly downregulated compared with the control cells (**Figure 4A**).

To verify whether USP22 mediates cisplatin resistance by regulating Ku70 acetylation via Sirt1, we inhibited the expression of Sirt1 while overexpressing USP22. Three pairs of siRNA named Sirt1-siRNA-1, Sirt1-siRNA-2, and Sirt1-siRNA-3. Compared with control, the expression level of Sirt1 was only decreased successfully by Sirt1-siRNA-2. The results revealed that, after the depletion of Sirt1 expression, Ku70 acetylation levels, cytochrome C level and the apoptosis rate increased significantly, and the Bax level binding to Ku70 decreased (**Figures 4B,C**), compared with that in A549-USP22 cells. To further confirm that USP22 induces cisplatin resistance via Sirt1, we added flow cytometric analysis results. After the depletion of USP22 or Sirt1 expression in A549/CDDP cells, the reverse effect to cisplatin resistance was stronger in sh-USP22 group than si-Sirt1 group, and there was no difference between sh-USP22+si-Sirt1 group and sh-USP22 group (**Figure 4D**). These results suggest that USP22 decreases the acetylation of Ku70 by stabilizing Sirt1, thus inhibiting Bax-mediated apoptosis and inducing cisplatin resistance.

### The Cisplatin Sensitivity in Cisplatin-Resistant A549/CDDP Cells Was Restored by USP22 Inhibition

**FIGURE 5:**
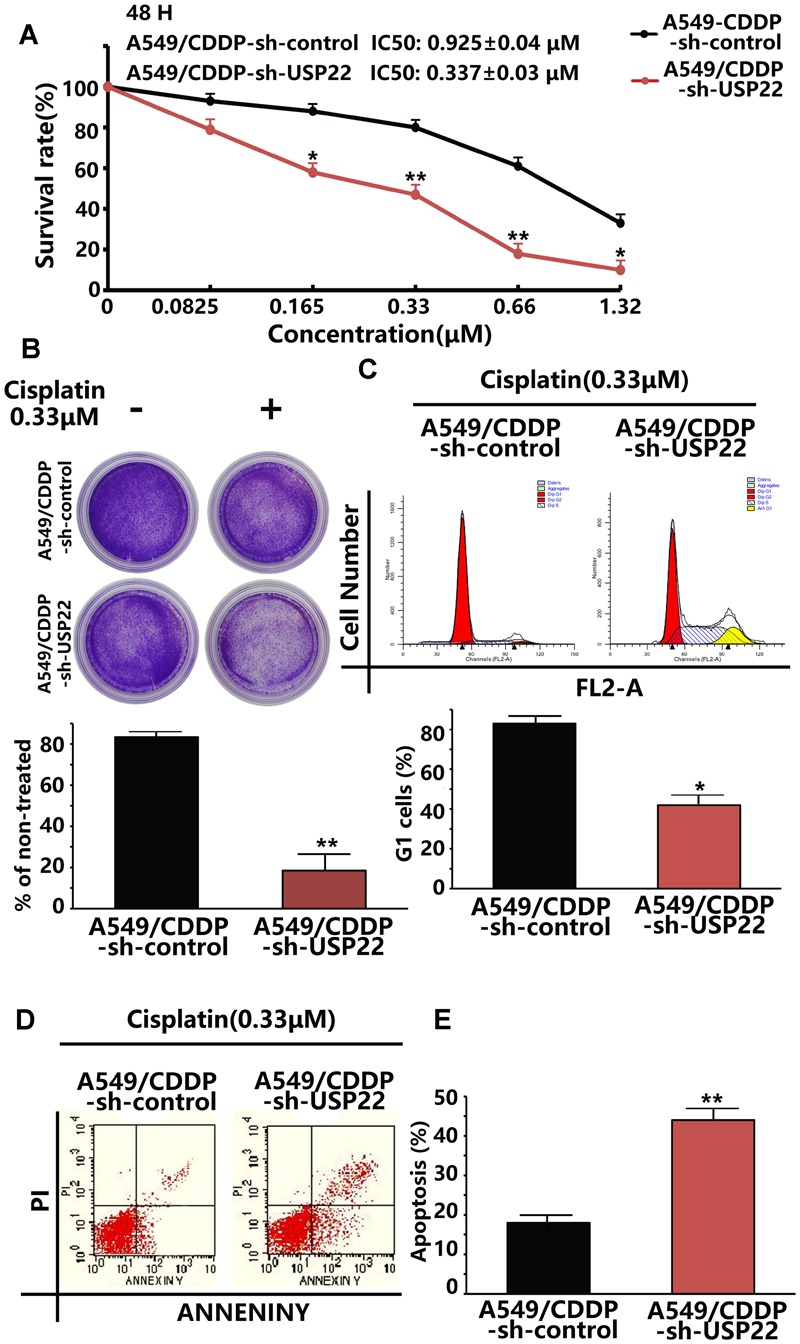
**The cisplatin sensitivity in cisplatin-resistant A549/CDDP cells was restored by USP22 inhibition. (A)** The sensitivity to cisplatin of A549/CDDP and A549/CDDP-sh-USP22 (the cell clones which were stably transfected with sh-USP22) cells was detected by CCK-8. Cells were exposed to various concentrations of cisplatin for 48 h. **(B)** The response of A549/CDDP and A549/CDDP-sh-USP22 cells to cisplatin was tested by colony-forming assay. Cell lines were treated continuously with 0 or 0.33 μM cisplatin for 14 days. **(C)** A549/CDDP and A549/CDDP-sh-USP22 cells were treated with 0.33 μM of cisplatin for 48 h and subjected to flow cytometry analysis of cell cycle distribution. Percentage change in G1 phase is shown. **(D)** Cisplatin-induced apoptosis in A549/CDDP and A549/CDDP-sh-USP22 cells was demonstrated by flow cytometric analysis. Cell lines were treated continuously with 0.33 μM of cisplatin for 48 h. **(E)** Every experiment was conducted at least three times, and the average is shown (mean ± SD). ^∗^*P* < 0.05, ^∗∗^*P* < 0.01, significant.

To verify whether inhibition of USP22 expression could reverse the cisplatin resistance of A549/CDDP cells, CCK8 assays showed that, after the inhibition of USP22 expression, the 48h IC50 of A549/CDDP decreased from 0.925 ± 0.04 μM to 0.337 ± 0.03 μM (**Figure 5A**). In addition, colony formation assays after cisplatin treatment showed that inhibiting USP22 expression led to a significant decrease in colony formation (**Figure 5B**). The subsequent flow cytometry analysis of cell cycle and apoptosis found that, after cisplatin treatment, the A549/CDDP-sh-USP22 cells showed significant G1 phase arrest and significantly increased apoptosis compared with the A549/CDDP-sh-control cells (**Figures 5D,E**). These results indicate that the cisplatin sensitivity in cisplatin-resistant A549/CDDP cells was restored by USP22 inhibition.

### Inhibiting USP22 Expression Enhanced Cisplatin Sensitivity in Cisplatin-Resistant A549/CDDP Cell Nude Mouse Xenografts

**FIGURE 6:**
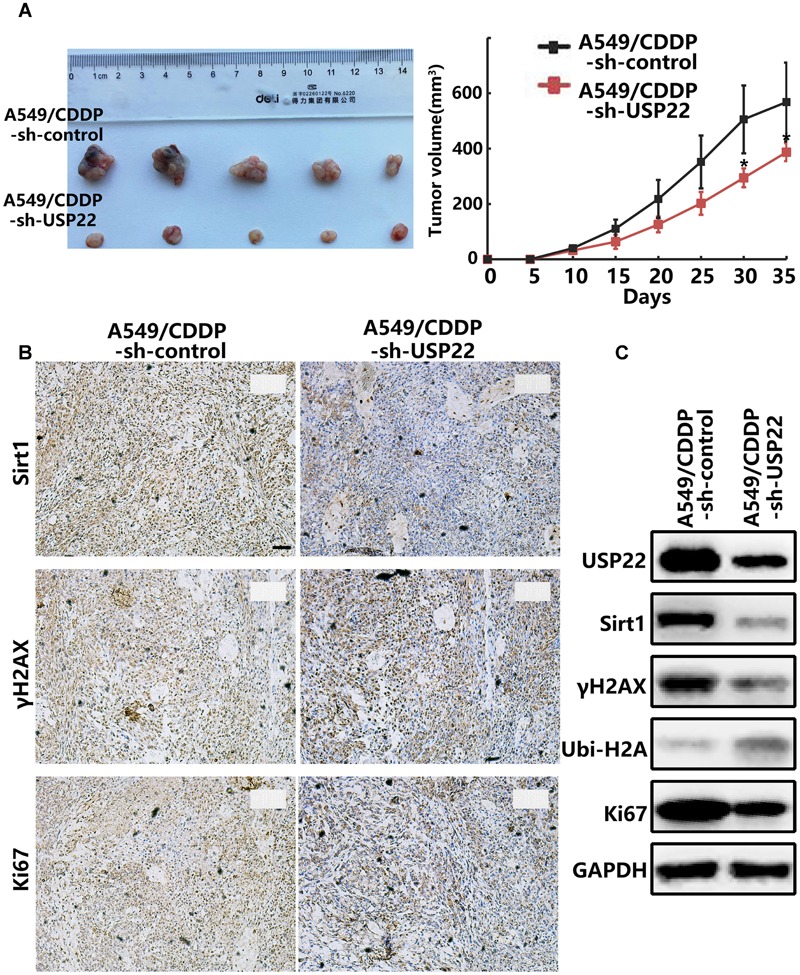
**Inhibiting USP22 expression enhanced cisplatin sensitivity in cisplatin-resistant A549/CDDP cell nude mouse xenografts. (A)** Representative figures and growth curve of tumor by subcutaneous injection of A549/CDDP and A549/CDDP-sh-USP22 cells. **(B)** IHC staining of Sirt1, γH2AX and Ki67 in tumor nodules from A549/CDDP and A549/CDDP-sh-USP22 cells derived xenografts. Bar: 200 μm. **(C)** Western blot analysis of USP22, Sirt1, γH2AX, Ubi-H2A, and Ki67 in A549/CDDP and A549/CDDP-sh-USP22 cells derived xenografts. Every experiment was conducted at least three times, and the average is shown (mean ± SD). ^∗^*P* < 0.05, ^∗∗^*P* < 0.01, significant.

A nude mouse xenograft model was established using A549/CDDP-sh-USP22 and A549/CDDP-sh-control cells to verify our previous conclusions *in vivo*. Tumors were formed in both groups of nude mice within 6–7 days. Both groups of nude mice received intra-peritoneal injection of cisplatin (3.5 mg/kg, thrice weekly injection [tiw]). The tumor volume of the A549/CDDP-sh-USP22 group was stably inhibited was significantly lower than the control group (*P* < 0.05) (**Figure 6A**). After 35 days, the mice were sacrificed, and the tumor tissues were removed and embedded in paraffin. Subsequent immunohistochemistry experiments showed that the expression of Sirt1, γH2AX and Ki67 in the nuclei was significantly lower in A549/CDDP-sh-USP22 group than the control group (**Figure 6B**). Western blots indicated that the expression levels of USP22, Sirt1, γH2AX, and Ki67 level decreased significantly and the ubiquitination of H2AK119 upregulated simultaneously in A549/CDDP-sh-USP22 group compared with the control group (**Figure 6C**). These results suggest that inhibiting USP22 expression enhanced cisplatin sensitivity in lung adenocarcinoma by downregulation of Sirt1 and γH2AX *in vivo*.

## Discussion

Systemic treatment fails in most of the NSCLC patients because of the rapid emergence of acquired resistance to platinum-based chemotherapy. Resistance to chemotherapeutic agents can involve various intrinsic cellular processes including drug efflux, increased resistance to apoptosis, or increased DNA repair capabilities in the case of platinum salts or other DNA damaging drugs. Herein, we focused our research on the involvement of the increased resistance to apoptosis and DNA repair processes in cisplatin resistance.

USP22, a novel deubiquitinating enzyme and a member comprising the 11 gene Polycomb/cancer stem cell signature, possesses the functions such as transcription activation, epigenetic regulation and cancer progression (Glinsky et al., 2005; Glinsky, 2006, 2008). Recent studies reported that overexpression of USP22 may be involved in the drug resistance of cancer through a variety of mechanisms. Our published research has demonstrated that USP22 was related to the chemotherapy resistance to gemcitabine in pancreatic cancer (Liang et al., 2014). A recent study suggested that cisplatin can suppress the expression of USP22 through p38/MAPK pathway in HeLa cells (Ding et al., 2014). USP22 can deubiquitinate Sirt1 and enhance its stability through c-MYC-related network, leading to FLT3 tyrosine kinase inhibitors (TKIs) resistance in acute myeloid leukemia (AML) (Li et al., 2014). USP22 controls androgen receptor (AR) accumulation and signaling, and is a predictor of androgen deprivation therapy in prostate adenocarcinoma (Schrecengost et al., 2014). USP22 acts as a major transcriptional factor to regulate major vault protein (MVP) drug resistant gene and links to chemotherapeutic failure in colorectal cancer (Xu et al., 2012). However, is USP22 also involved in the process of cisplatin resistance in lung adenocarcinoma? In the present study, we observed that upregulation of USP22 could induce cisplatin resistance in A549 cells (**Figures 1B,C**), and the level of USP22 was upregulated significantly in cisplatin-resistant A549/CDDP cells (**Figure 2A**). Cisplatin is a cell-cycle non-specific cytotoxic drug and leads to G1-S arrest (Maccio and Madeddu, 2013). We observed that the upregulation of USP22 could overcome the G1-S phase arrest induced by cisplatin (**Figure 1D**) and inhibit apoptosis (**Figure 1E**). Combining with our results of CCK8 assay and flow cytometric analysis in A549-USP22 and A549-CDDP cells, we conclude that in the same condition of cisplatin concentration, the tumorigenesis abilities of A549-CDDP cells is higher than A549-USP22 cells. Therefore, these results suggest that USP22 is one of the mechanisms of cisplatin resistance but not all.

Cisplatin is a heavy metal complex in which divalent platinum is combined with two chlorine atoms and two ammonia molecules, and it acts like a bifunctional alkylating agent and inhibits DNA replication. DNA repair is one of the main mechanisms underlying cisplatin resistance. The DNA DSB is the most dangerous form of DNA damage caused by cisplatin (Galluzzi et al., 2014). Our study showed that USP22 could significantly reduce the frequency of DNA DSBs induced by cisplatin (**Figure 3A**). USP22 is localized in the nucleus and is capable of deubiquitinating histones H2A and H2B (Lang et al., 2011). In this study, we verified that USP22 bind to endogenous H2A in A549 cell line by IP (**Figures 3B,C**). Histone H2A is also mono-ubiquitinated at Lys119 at sites of DNA damage. Histone H2AX is a subtype of histone H2A. In the process of DSB repair, the H2AX 139^Ser^site is phosphorylated to form γH2AX. Histone H2AX phosphorylation on γH2AX is a sensitive marker for DNA DSBs (Rogakou et al., 1999). It has been reported that γH2AX is associated with resistance to radiation and multiple chemotherapy drugs including cisplatin (Adam-Zahir et al., 2014; van Oorschot et al., 2016). Our study suggested that, the level of γH2AX was upregulated in cisplatin-resistant A549/CDDP cells (**Figure 2B**) and increased significantly after cisplatin treatment (**Figure 2C**). After cisplatin treatment, the levels of γH2AX and H2AX binding to USP22 increased significantly, and the ubiquitination of H2A decreased simultaneously in A549-USP22 and A549/CDDP cells (**Figure 3D**). These findings confirm that USP22 could deubiquitinate H2AX and promote its phosphorylation, thus contributing to DNA damage repair and inducing cisplatin resistance.

In addition, cisplatin can induce cell death by engaging endogenous apoptotic signaling that activates mitochondrial apoptosis (Galluzzi et al., 2012). The mitochondrial pathway of apoptosis is mainly regulated by mitochondria-related Bcl-2 family members, such as Bcl-2 and Bax. Multi-domain pro-apoptotic Bax is an executor of apoptosis and its oligomerizations on the outer mitochondrial membrane is required for the release of cytochrome C and induction of apoptosis. Loss of Bax leads to complete resistance to cisplatin, due to failure in the induction of mitochondrial outer membrane permeabilization (MOMP) and subsequent apoptotic events (Wang and Youle, 2012). Ku70, a DNA repair protein, has recently been shown to suppress apoptosis by sequestering Bax from mitochondria (Sawada et al., 2003). Previous studies indicate a positive relationship between Ku70 and cancer progression, genotoxic and chemotherapy resistance (including cisplatin), highlighting Ku70-Bax interaction as an important therapeutic target of reversing resistance (Gullo et al., 2006; Shukla et al., 2014). The interaction between Bax and Ku70 is also regulated by an acetylation-dependent mechanism. We observed that the level of Ku70 acetylation and cytochrome C in A549/CDDP cells and A549-USP22 cells was significantly downregulated, whereas the level of Bax binding to Ku70 was significantly upregulated (**Figure 4A**). Sirt1 is a NAD^+^-dependent protein deacetylase that deacetylates histones including H4K16 to regulate gene expression and many non-histone proteins for biological functions (George and Ahmad, 2016). We found that Sirt1 levels were significantly increased in A549-USP22 and A549/CDDP cells. Deacetylation of Ku70 by Sirt1 sequesters Bax protein in the cytoplasm to prevent apoptosis initiation and extend cell survival (Cohen et al., 2004). A recent report showed that Sirt1 expression was a strong predictor for poor prognosis in NSCLC patients underwent platinum-based chemotherapy, and interfering with Sirt1 expression significantly enhanced the chemosensitivity to cisplatin treatment (Zhang et al., 2013). USP22 is reported to deubiquitinate and stabilize Sirt1, participating in processes regulated by Sirt1 acetylation such as cell proliferation, apoptosis and DNA damage repair. Our study suggested that, the level of Sirt1 was upregulated in cisplatin-resistant A549/CDDP cells (**Figure 2B**) and increased significantly after cisplatin treatment (**Figure 2C**). Ku70 acetylation levels, the Bax level binding to Ku70, the cytochrome C level (**Figure 4B**) and the apoptosis rate (**Figure 4C**) increased significantly after knockdown of Sirt1 while overexpressing USP22. After the depletion of USP22 or Sirt1 expression in A549/CDDP cells, the reverse effect to cisplatin resistance was stronger in sh-USP22 group than si-Sirt1 group, and there was no difference between sh-USP22+si-Sirt1 group and sh-USP22 group (**Figure 4D**). Combining with our previous results, we concluded that both USP22 and Sirt1 can induce cisplatin resistance, but Sirt1 overexpression can’t phenocopy USP22-mediated cisplatin resistance. Moreover, the resistance degree of USP22-mediated cisplatin resistance was higher than Sirt1-mediated cisplatin resistance. These results suggest that USP22 decreases the acetylation of Ku70 by stabilizing Sirt1, thus inhibiting Bax-mediated apoptosis and inducing cisplatin resistance.

To our knowledge, this is the first report to link USP22 with cisplatin resistance in lung adenocarcinoma. Our findings in lung adenocarcinoma cell line (**Figure 5**) and xenografts (**Figure 6**) support that USP22 could promote the phosphorylation of histone H2AX via deubiquitinating histone H2A, thus contributing to the DNA damage repair induced by cisplatin and leading to cisplatin resistance. In addition, USP22 could also decrease the acetylation of Ku70 by stabilizing the expression of Sirt1, thus inhibiting Bax-mediated apoptosis and contributing to cisplatin resistance. The cisplatin sensitivity in cisplatin-resistant A549/CDDP cells was restored by USP22 inhibition *in vivo* and *vitro*. Interestingly, these results reveal the dual mechanism of USP22 involvement in cisplatin acquired resistance and inhibiting USP22 expression could enhance cisplatin sensitivity in lung adenocarcinoma. Previous studies have confirmed that USP22 is significantly upregulated and associated with therapy failure and poor prognosis in several solid tumors, including NSCLC. Therefore, USP22 maybe also participate in the intrinsic resistance of cisplatin, but this conclusion remains to be confirmed in further experiments. In conclusion, USP22 is a potential target in cisplatin-resistant lung adenocarcinoma and should be considered in future therapeutic practice.

## Conclusion

Our study confirmed that the overexpression of USP22 was correlated with acquired resistance to cisplatin in lung adenocarcinoma. The study reveal the dual mechanism of USP22 involvement in cisplatin resistance: (1) USP22 enhances DNA damage repair and induce cisplatin resistance by promoting the phosphorylation of histone H2AX via deubiquitinating histone H2A. (2) USP22 decreases the acetylation of Ku70 by stabilizing Sirt1, thus inhibiting Bax-mediated apoptosis and inducing cisplatin resistance. In addition, the cisplatin sensitivity in cisplatin-resistant A549/CDDP cells was restored by USP22 inhibition *in vivo* and *vitro*. Our findings suggest that USP22 is a potential target in cisplatin-resistant lung adenocarcinoma and should be considered in future therapeutic practice.

## Author Contributions

AW, ZN, GT, and JwL designed the research. QY, GT and JwL conducted the all experiments. AW, ZN, CL, WG, and JxL performed data analysis. All authors contributed to the writing and final approval of the manuscript.

## Conflict of Interest Statement

The authors declare that the research was conducted in the absence of any commercial or financial relationships that could be construed as a potential conflict of interest.
